# Neural Markers of Auditory Response and Habituation in Phelan-McDermid Syndrome

**DOI:** 10.3389/fnins.2022.815933

**Published:** 2022-05-03

**Authors:** Emily L. Isenstein, Hannah E. Grosman, Sylvia B. Guillory, Yian Zhang, Sarah Barkley, Christopher S. McLaughlin, Tess Levy, Danielle Halpern, Paige M. Siper, Joseph D. Buxbaum, Alexander Kolevzon, Jennifer H. Foss-Feig

**Affiliations:** ^1^Department of Brain and Cognitive Sciences, University of Rochester, Rochester, NY, United States; ^2^Department of Psychological and Brain Sciences, Drexel University, Philadelphia, PA, United States; ^3^Seaver Autism Center, Icahn School of Medicine at Mount Sinai, New York, NY, United States; ^4^Department of Psychiatry, Icahn School of Medicine at Mount Sinai, New York, NY, United States; ^5^Center for Neural Science, New York University, New York, NY, United States; ^6^Mindich Child Health and Development Institute, Icahn School of Medicine at Mount Sinai, New York, NY, United States; ^7^Department of Neuroscience, Icahn School of Medicine at Mount Sinai, New York, NY, United States; ^8^Department of Genetics and Genomic Sciences, Icahn School of Medicine at Mount Sinai, New York, NY, United States; ^9^Department of Pediatrics, Icahn School of Medicine at Mount Sinai, New York, NY, United States

**Keywords:** phelan-mcdermid syndrome, autism spectrum disorder, EEG, auditory, perception, habituation, sensory, genetics

## Abstract

Phelan-McDermid Syndrome (PMS) is a rare genetic disorder caused by deletion or sequence variation in the *SHANK3* gene at terminal chromosome 22 that confers high likelihood of comorbid autism spectrum disorder (ASD). Whereas individuals with idiopathic ASD (iASD) can demonstrate diverse patterns of sensory differences, PMS is mainly characterized by sensory hyporesponsiveness. This study used electrophysiology and a passive auditory habituation paradigm to test for neural markers of hyporesponsiveness. EEG was recorded from 15 individuals with PMS, 15 with iASD, and 16 with neurotypical development (NT) while a series of four consecutive 1,000 Hz tones was repeatedly presented. We found intact N1, P2, and N2 event-related potentials (ERPs) and habituation to simple auditory stimuli, both in individuals with iASD and in those with PMS. Both iASD and PMS groups showed robust responses to the initial tone and decaying responses to each subsequent tone, at levels comparable to the NT control group. However, in PMS greater initial N1 amplitude and habituation were associated with auditory hypersensitivity, and P2 habituation correlated with ASD symptomatology. Additionally, further classification of the PMS cohort into genetic groupings revealed dissociation of initial P2 amplitude and habituation of N1 based on whether the deletions included additional genes beyond solely *SHANK3* and those not thought to contribute to phenotype. These results provide preliminary insight into early auditory processing in PMS and suggest that while neural response and habituation is generally preserved in PMS, genotypic and phenotypic characteristics may drive some variability. These initial findings provide early evidence that the robust pattern of behavioral hyporesponsiveness in PMS may be due, at least in audition, to higher order factors.

## Introduction

Phelan-McDermid syndrome (PMS) is a rare neurodevelopmental disorder caused by haploinsufficiency of *SHANK3* either by pathogenic sequence variant or by deletion (Phelan and McDermid, 2012; Oberman et al., 2015). PMS is characterized by global developmental delay, absent or delayed speech, hypotonia, and dysmorphic features (Soorya et al., 2013). Comorbid autism spectrum disorder (ASD) is common in PMS, with up to 84% of individuals receiving a diagnosis (Soorya et al., 2013), though estimates vary substantially (Phelan and McDermid, 2012; Sarasua et al., 2014). Both PMS and ASD are associated with sensory reactivity differences. A recent study showed that the sensory reactivity symptoms associated with PMS are distinct from those typically associated with ASD, wherein individuals with PMS have greater hyporeactivity and fewer hyperreactivity and sensory seeking symptoms compared to individuals with idiopathic ASD (iASD) and typically developing controls (Tavassoli et al., 2021). Evidence of hyporeactivity also comes from clinical reports of many individuals with PMS displaying delayed response to auditory and verbal cues, despite having no hearing impairments (Phelan and McDermid, 2012). However, research to date has heavily relied on caregiver report.

Electroencephalography (EEG) offers a precise and objective tool to evaluate sensory processing. One method of assessing auditory perception is by measuring habituation, or the decrease in electrophysiological activity in response to repeatedly presented sounds. In neurotypical individuals (Fruhstorfer et al., 1970) and in animal models (Sambeth et al., 2004), this brain response is consistent: to the first tone in sequence, a large response is elicited; thereafter, the response is dampened with the strongest decline between the first and second repetitions (Budd et al., 1998; Ozesmi et al., 2000). Habituation paradigms have been used in ASD research on several occasions to explore auditory sensory differences but have yielded mixed results. Some studies found slower habituation to auditory stimuli in ASD (Ornitz et al., 1993; Hudac et al., 2018; Green et al., 2019; Jamal et al., 2021), while others found no differences between ASD and neurotypical (NT) children or adults (Kohl et al., 2014; Takahashi et al., 2016). These findings showcase the heterogeneity found within ASD and emphasize the difficulty in establishing unifying, biologically-based characteristics in the absence of stratifying variables.

A small body of research has used electrophysiology as an objective measure of auditory responsiveness in people with genetic disorders related to ASD, though not yet in PMS. For example, studies of auditory processing have identified atypical electrophysiologic signatures in neurodevelopmental disorders including Rett syndrome (Sysoeva et al., 2020) and Tuberous Sclerosis Complex (O’Brien et al., 2020). Notably, one study used a habituation paradigm in Fragile X syndrome (FXS), recording cortical activity during passive listening to repeated sequences of identical auditory tones. This study successfully captured electrophysiological evidence for cortical hyper-excitability in individuals with FXS that also correlated with parental reports of sensory sensitivity (Ethridge et al., 2016). This study’s recapitulation of findings in *Fmr1* knockout mice (Frankland et al., 2004) suggests that similar parallels may exist between humans with PMS and *Shank3* rodent models.

Although no auditory electrophysiology work has been undertaken in humans with PMS to date, work in animal models provides clues to the underlying neurophysiology and expected phenotype. A recent manuscript utilized a *Shank3* mouse model and found reduced startle response across a variety of sound intensities (Drapeau et al., 2018). These mice showed normal Preyer reflexes, indicating aberrations in auditory processing rather than broader issues with sensory gating. Another study found weaker electrophysiological responses and decreased levels of spontaneous firing in the auditory cortex in *Shank3* heterozygous rats compared to wild-type rats (Engineer et al., 2018). These rodents also showed a decreased number of spikes evoked in response to noise burst trains, with the responses to the successive rapid noise burst showing the greatest reduction. Together, these results suggest that *Shank3* deficiency in animals confers a delayed and less vigorous cortical response to auditory stimuli, but this biomarker has not yet been translated to humans with PMS. Such translational work would not only offer the opportunity to more deeply understand the neurobiological alterations identified in patients, but also serve as biomarkers for potential treatment approaches that could be tested in animal models then brought back to human patients.

To bridge the gaps between pre-clinical and clinical work, the current study tested electrophysiological correlates of auditory response and habituation in individuals with PMS, as compared to those with iASD and NT controls. In addition, the present study examined the relation between EEG markers of auditory function and several clinical indices, including extent of 22q13 deletion, age, developmental quotient, autism diagnosis, and sensory symptoms. By investigating whether behavioral auditory hyposensitivity has detectable electrophysiologic correlates, we hoped to identify whether changes in early cortical processing drive this phenotype. If people with PMS display blunted electrophysiological responses to new or repeated sounds, it would implicate early sensory processing as the source of dysfunction. On the other hand, if electrophysiological habituation is not disrupted in PMS, this suggests alternative, perhaps higher level or more domain-general, parts of the sensory-perceptual pathway as the drivers of behavioral alterations. Based on both the clinical phenotype in PMS and on animal findings, we predicted that, as compared to the NT group, the PMS group would have smaller amplitude responses and longer latencies to initial tones, followed by weaker habituation over time. We also predicted that, compared to the NT group, the iASD group would have similar latencies to initial tones but habituate more slowly over time and have overall higher amplitudes, based on the trait of auditory hypersensitivity that many iASD individuals share with individuals with FXS. Finally, we also expected that clinician and parent reports of sensory hyporeactivity within the PMS group would correlate with reduced amplitude and habituation of electrophysiological components.

## Materials and Methods

### Participants

Written informed consent was obtained from all participants or their caregivers, as appropriate, and verbal assent was obtained from all participants under the age of 18 who were able to provide it, as approved by the Icahn School of Medicine Program for the Protection of Human Subjects.

Participants included 15 individuals with PMS (Mean = 14.7 years, SD = 6.4), 15 with iASD (Mean = 14.3 years, SD = 5.6), and 16 with NT development (Mean = 13.1 years, SD = 4.3), all between the ages of 8 and 26. The groups did not differ significantly in age [*F*(2, 43) = 0.32, *p* = 0.73]. A Chi Square analysis also identified no significant sex differences among groups [*X*^2^ (2, *n* = 46) = 5.51, *p* = 0.06]. In the PMS group, chromosomal microarray or targeted sequencing of the *SHANK3* gene validated the genetic diagnosis. Confirmed genetic diagnoses of PMS were defined as having either a deletion encompassing *SHANK3* (MIM: 606230) or a pathogenic sequence variant in *SHANK3* according to standards established by the American College of Medical Genetics and Genomics and the Association for Molecular Pathology. Microarray results were aligned to the hg19 reference genome and sequence variants to reference transcript NM_033517.1. Of the individuals with PMS, 11 had deletions encompassing *SHANK3* and four had pathogenic sequence variants in *SHANK3*. One of the individuals with a deletion had a ring chromosome 22. Participants in the iASD group had no known pathogenic genetic findings. Neurotypical controls had no psychiatric disorders and no first-degree relatives with iASD.

Phelan-McDermid Syndrome and iASD participants received autism diagnostic testing with the Autism Diagnostic Observation Schedule - 2 (ADOS-2, Lord et al., 2012) administered by a trained, research-reliable clinician. ADOS-2 social affect scores, restricted and repetitive behavior scores, and total scores did not differ between clinical groups (see Table 1), with 1 participant with iASD missing ADOS-2 data. The Autism Diagnostic Interview - Revised (ADI-R, Lord et al., 1994) was administered to the PMS group by a trained, research-reliable clinician to more deeply and precisely characterize developmental history of ASD symptoms, as this cohort is particularly hard to study with standardized assessments given the level of intellectual disability and other comorbidities. Clinical consensus among licensed psychiatrists and clinical psychologists confirmed final diagnoses with 33% of the PMS group receiving an ASD diagnosis. Sensory symptomatology in the PMS group was further characterized using the Sensory Assessment for Neurodevelopmental Disorders (SAND, Siper and Tavassoli, 2021), which incorporates clinician-administered observation with caregiver interview to measure sensory symptoms associated with DSM-5 criteria for ASD, and has been validated in both ASD and PMS (Siper et al., 2017; Tavassoli et al., 2021). SAND data was missing for one participant with PMS.

**TABLE 1:** Clinical and demographic information.

	Group Mean (SE)				
	PMS (*n* = 15)	iASD (*n* = 15)	NT (*n* = 16)	Statistic	*p*-Value
Age (year)	14.99 (6.61)	15.08 (0.81)	13.66 (4.38)	*F* = 0.32	0.73
Sex (M/F)	8M, 7F	11M, 4F	5M, 11F	*X*^2^(2) = 5.51	0.06
Cognitive level	36.47 (16.77)	97.02 (27.51)		*t* = 7.21	*p* < 0.001
**ADOS-2**					
Social affect	10.92 (0.90)	10.20 (1.51)	–	*t* = -0.39	0.70
Restricted and repetitive behaviors	4.08 (0.85)	3.13 (0.39)	–	*t* = -1.06	0.30
Total	13.36 (1.35)	13.33 (1.83)	–	*t* = -0.01	0.99
**ADI-R domain scores**					
Language/communication	10.00 (1.40)				
Reciprocal social interaction	13.80 (2.42)				
Restricted and repetitive behaviors	3.80 (0.63)				

Intelligence Quotient (IQ) testing appropriate for age and developmental functioning was administered to the iASD and PMS groups, including the Wechsler Abbreviated Scale of Intelligence – Second Edition (Wechsler, 2011), Stanford-Binet Intelligence Scales – Fifth Edition (Roid, 2003), Mullen Scales of Early Learning (Mullen, 1995), Differential Ability Scales – Second Edition (Elliott, 2007), and Wechsler Intelligence Scale for Children – Fifth Edition (Wechsler et al., 2014). Five of 15 PMS participants did not meet basal threshold requirements to receive an IQ score, so developmental quotients [DQ: (mental age/chronological age) × 100] scores were computed from subtest-level age equivalent scores to provide a standardized DQ (ratio IQ) measure used in place of IQ, allowing us to compare data across the various IQ instruments. There was a significant difference in cognitive level between iASD and PMS groups [*t*(2, 27) = 7.21, *p* < 0.001]. One iASD participant was missing an IQ score. IQ testing was not done with the NT group, but none had history of learning, psychiatric, or neurodevelopmental disorders or concerns, thus we estimate their IQs to have followed a typical normative distribution and therefore also to be higher than both iASD and PMS groups.

### Experimental Procedure

Participants completed a 16-min auditory habituation task during dense-array EEG, while seated in a chair, booster seat, or caregiver’s lap, as best facilitated their remaining seated and still. Each of 150 trials contained a sequence of four 1,000 Hz, 50 ms tones (generated in Audacity), separated by 618 ms. Trials were separated by a 4,000 ms inter-stimulus-interval. Experimental flow was controlled using E-Prime 2.0. Tones were delivered at 80 dB. Participants were not instructed to attend to the tones, and each watched a silent video of their choice throughout the duration of the experiment.

### Electroencephalography Data Acquisition and Analysis

Continuous EEG data were collected using a 128-channel Philips HydroCel Geodesic Sensor Net and NetStation Software Version 5.3. Data were re-referenced to average reference and high-pass filtered at 0.5 Hz. Data were then processed within Matlab using the Fully Automated Statistical Thresholding for EEG artifact Rejection (FASTER) Routine toolbox (Nolan et al., 2010) with EEGlab (Delorme and Makeig, 2004). The FASTER routine employs multiple measures for identifying statistical outliers within the data. Continuous EEG data were segmented into 3,000 ms epochs from −1,500 to 1,500 ms, and time-locked to the onset of each tone during FASTER pre-processing. The processing steps involve classifying and replacing outlier channels with interpolated values in the continuous data, removing outlier epochs from single participant data, removing outlier components through spatial independent component analysis, and correcting outlier channels by interpolating single channels within a single epoch. All participants were presented the same total of 600 tones (150 sets of 4). The NT, PMS, and iASD groups had averages of 3.95 ± 0.96, 3.17 ± 1.23, and 3.47 ± 1.12% trials removed, respectively. The groups did not vary significantly by proportion of trials removed [*F*(1,2) = 1.97, *p* = 0.15]. Lastly, ERPs were averaged separately for each of the four tones in the trial’s sequence and baseline corrected using a 100 ms pre-stimulus interval. A low-passed filter of 30 Hz was used to account for noise caused by considerable amounts of movement by some of the PMS and iASD participants. This filter level is higher than (Sysoeva et al., 2020) or consistent with (O’Brien et al., 2020) other research on auditory processing in genetic conditions. ERP averages were computed using a trimmed means method, discarding the top and bottom 5% of data from each time point to yield a robust mean estimate (Leonowicz et al., 2005).

#### Event-Related Potentials Analysis

N1, P2, and N2 mean amplitudes were calculated as the individual averages of the 10 ms surrounding each peak at electrode Cz at the vertex of the head, where auditory event related potential are easily detected (Rosburg et al., 2010). For each participant, the P2 component was first identified in the ERP as the maximum voltage within the latency range 120–210 ms post-stimulus onset. Next, using the computed P2 latency value, the N1 component was defined as the minimum voltage within the window from 70 ms until the P2 time point. The N2 peak amplitude was quantified as the minimum voltage in the time window that started at the computed P2 latency until 385 ms. Latency was defined as time to peak amplitude for each component.

Statistical analysis was done using SPSS software and included one-way ANOVAs to compare the effect of group on individual component amplitude and latency within each tone. Additionally, repeated measures ANOVAs were conducted to compare habituation of component amplitude across the four tones by group. Greenhouse-Geiser was used when there were violations in sphericity. When Levene’s Test was significant, the Brown-Forsythe results or the combination of Friedman and Kruskal-Wallis Tests were used, as appropriate. Correlations were run between all clinical and electrophysiological variables to assess relationships. Bonferroni corrections for multiple comparisons were used in the analyses, with a significant *p*-value set at 0.008 for the initial tone analysis and 0.002 for analyses of habituation across tones.

## Results

### Response to Initial Tone

**FIGURE 1:**
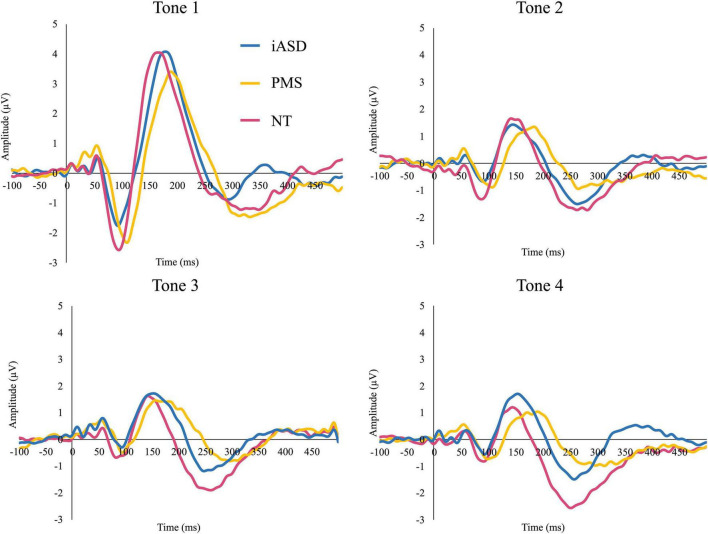
Event-related potentials (ERP) response to consecutive four tones in individuals with idiopathic ASD (iASD), Phelan-McDermid Syndrome (PMS), and neurotypical development (NT) groups.

**FIGURE 2:**
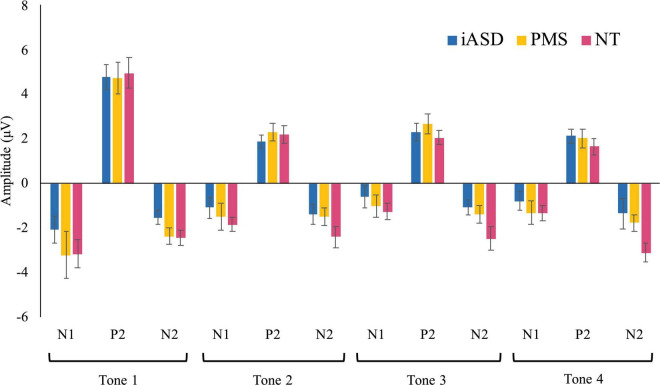
Means of each ERP component in response to tones 1–4 with standard error bars in iASD, PMS, and NT groups.

Applying three separate one-way ANOVAs, one for each component, analysis of the ERP response to the initial tone found peak amplitude was not significantly altered across diagnostic groups (see Figures 1, 2 and Table 2; all *p*’s > 0.13, all η^2^*_*p*_* < 0.09). Three one-way ANOVAs were conducted to determine group differences between latency values for each component in response to the initial tone, again with no significant differences among groups (see Table 2; all *p*’s > 0.11, all η^2^*_*p*_* < 0.12).

**TABLE 2:** Analysis of group differences to initial tone.

	Group mean (SE)			Statistics	
	PMS	iASD	NT	*F* Statistic	*p*-value
***Initial tone***					
N1 amplitude	−2.87 (1.00)	−1.81 (0.62)	−2.77 (0.62)	*F* = 0.57	0.57
P2 amplitude	4.53 (0.71)	4.60 (0.52)	4.68 (0.68)	*F* = 0.02	0.98
N2 amplitude	−2.49 (0.30)	−1.64 (0.18)	−2.29 (0.34)	*F* = 2.13	0.13
N1 latency	207.20 (6.26)	193.38 (2.65)	197.75 (3.61)	*F* = 2.42	0.11
P2 latency	298.53 (11.90)	276.69 (5.56)	279.13 (6.66)	*F* = 2.02	0.14
N2 latency	413.73 (7.92)	395.06 (7.73)	410.38 (10.78)	*F* = 1.24	0.30

### Habituation of Amplitude Across Tones

To determine changes in the ERP waveform across the sequence of the four tones from habituation effects, amplitude of the N1, P2, and N2 were each subjected to a repeated measures ANOVA. Non-parametric tests, Friedman and Kruskal Wallis, were applied when violations of ANOVA assumptions were noted.

Analysis of the N1 found a significant effect of tone position [*F*(1.65, 70.99) = 13.64, *p* < 0.001, η^2^*_*p*_* = 0.50] where there was a marked reduction in amplitude between the 1st tone (N1_1_: −2.49 ± 0.44 μV) and the 2nd, 3rd, and 4th tones in the sequence (N1_2_: −1.25 ± 0.26 μV, *p* < 0.001, *d* = 0.57; N1_3_: −0.74 ± 0.26 μV, *p* < 0.001, *d* = 0.74; N1_4_: −0.95 ± 0.24 μV, *p* < 0.001, *d* = 0.68). Further habituation effects were also detected between the response to the N1_2_ tone and the N1_3_ tone (*p* = 0.003, *d* = 0.29), though the amplitude difference between the N1_2_ and N1_4_ amplitudes did not reach significance (*p* = 0.12, *d* = 0.20). There was no significant difference between the N1_3_ and N1_4_ amplitudes (*p* = 1.00, *d* = 0.11), suggesting response to repetition was maximally habituated by the third repetition. The effect of group [*F*(2,43) = 0.62, *p* = 0.54, η^2^*_*p*_* = 0.03] and the group by tone position interaction [*F*(3.30, 70.99) = 0.33, *p* = 0.83, η^2^*_*p*_* = 0.02] were not significant.

Similar to results for the N1 component, P2 amplitude showed a significant effect of tone position [*F*(3, 70.92) = 76.82, *p* < 0.001, η^2^*_*p*_* = 0.64], with a significantly larger P2_1_ amplitude (4.60 ± 0.37 μV) as compared to the subsequent P2_2_ (1.96 ± 0.22 μV, *d* = 1.30), P2_3_ (2.16 ± 0.22 μV, *d* = 1.20), and P2_4_ (1.76 ± 0.22 μV, *d* = 1.41) tones (all *p*’s < 0.001, all *d*’s > 1.20). Unlike for N1, P2_2_ amplitude did not differ from either P2_3_ (*p* = 1.00, *d* = 0.13) or P2_4_ (*p* = 0.94, *d* = 0.15) tones, but P2_3_ was larger in amplitude compared to P2_4_ (*p* = 0.03, *d* = 0.27). Again, there was no significant effect of group [*F*(2,44) = 0.09, *p* = 0.92, η^2^*_*p*_* < 0.01] and no significant tone position by group interaction [*F*(6,70.20) = 0.62, *p* = 0.62, η^2^*_*p*_* = 0.03].

Unlike the previous components, the effect of tone position on the N2 component did not withstand correction for multiple comparisons [*F*(2.45, 105.40) = 3.18, *p* = 0.04, η^2^*_*p*_* = 0.07]. All other comparisons were not significant, with no significant group effect [*F*(2,43) = 3.19, *p* = 0.05, η^2^*_*p*_* = 0.13] or group by tone position interaction [*F*(4.90, 105.40) = 1.45, *p* = 0.21, η^2^*_*p*_* = 0.06] for N2.

### Exploratory Analyses

**FIGURE 3:**
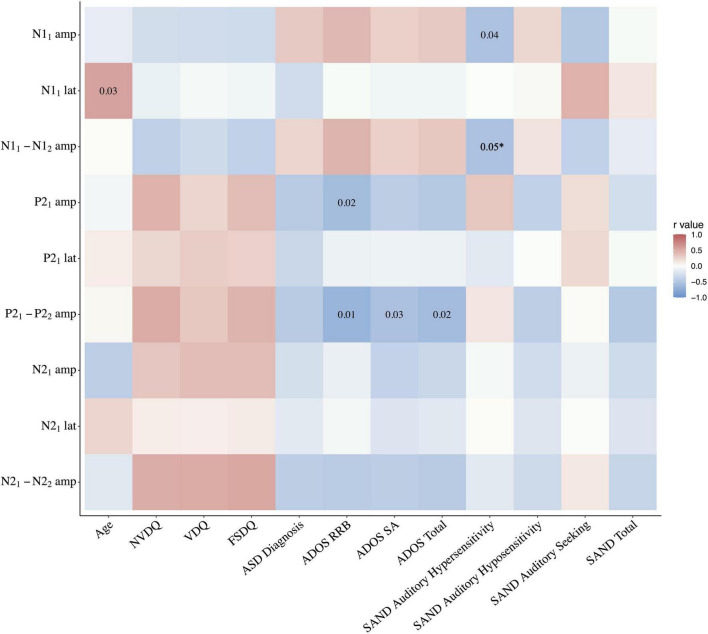
Correlation heatmap of electrophysiologic findings and clinical indices in the PMS group. *Correlation between SAND auditory hypersensitivity and N1_1–2_ habituation *p* = 0.048. amp, amplitude; lat, latency; NVDQ, Non-verbal Developmental Quotient; VDQ, Verbal Developmental Quotient; FSDQ, Full Scale Developmental Quotient; ADOS, Autism Diagnostic Observation Scale; RRB, Restricted and Repetitive Behaviors; SA, social affect; SAND, Sensory Assessment for Neurodevelopmental Disorders.

Correlation analyses were run for the PMS group with: (a) the mean amplitude and latency values for each component at tone 1, and (b) the difference between each component’s amplitude at tone 1 and tone 2, used as a proxy measure of habituation. For negative components N1 and N2, a greater negative number indicates greater habituation. Correlations were not corrected for multiple comparisons due to the stringent threshold criteria driven by the limited sample size. With so little known about sensory processing in PMS, we believe that applying overly strict standards would obscure potential avenues for future research and slow progression of knowledge in this field. As the first study of auditory electrophysiological correlates of auditory hyposensitivity in a rare and difficult to test population, these data are important to include but should be interpreted cautiously as exploratory findings. See Figure 3 for full statistics.

#### Genetics

Per the categorization scheme outlined in a recent manuscript on associations between genotype and phenotype in PMS, we divided participants with deletions into two classes (Levy et al., 2021). Class I was comprised of sequence variants as well as deletions including only *SHANK3*, or the combination of *SHANK3* with *ARSA* and/or *ACR* and *RABL2B* (*n* = 11); these final three genes are not thought to contribute to the phenotype of PMS. Class II was comprised of the remaining deletions that did not qualify as Class I deletions, i.e., larger deletions that extended beyond *SHANK3* and the three aforementioned genes (*n* = 4). Deletion size was not normally distributed so equal variance was not assumed.

The P2_1_ amplitude in Class I (5.16 ± 0.89) was greater than Class II (2.80 ± 0.47, *p* = 0.04, Δ = 0.95) and N1_1–2_ habituation was greater in Class I (−2.30 ± 0.66) compared to Class II (0.49 ± 0.62, *p* = 0.01, Δ = 1.24). No other electrophysiological measures differed between Classes. See Table 3.

**TABLE 3:** Analysis of PMS genetic classes and electrophysiological measures.

	Group mean (SE)		Statistics	
	Class I	Class II	*F* Statistic	*p*-Value
***Initial tone***				
N1 amplitude	−3.93	0.08	*F* = 4.58	0.07
P2 amplitude	5.16	2.80	*F* = 5.42	0.04
N2 amplitude	−2.29	−3.04	*F* = 2.51	0.14
N1 latency	209.10	202.00	*F* = 0.12	0.75
P2 latency	303.09	286.00	*F* = 0.29	0.62
N2 latency	416.18	407.00	*F* = 0.15	0.72
***Habituation***				
N1 amplitude	−2.30	0.49	*F* = 9.47	0.01
P2 amplitude	2.80	1.37	*F* = 2.38	0.16
N2 amplitude	−0.99	−1.31	*F* = 0.18	0.68

#### Age

Age did not correlate with any of the metrics specified above (*p*’s > 0.20).

#### Developmental Quotient

Non-verbal Developmental Quotient (NVDQ), Verbal Developmental Quotient (VDQ), and Full Scale Developmental Quotient (FSDQ) did not correlate with any ERP response or habituation metric (*p*’s > 0.05). See Figure 3.

#### Autism Spectrum Disorder Diagnosis

There were no significant Pearson correlations between clinical consensus ASD diagnosis and any of the metrics specified above (*p*’s > 0.09). See Figure 3.

#### Autism Diagnostic Observation Schedule - 2

**FIGURE 4:**
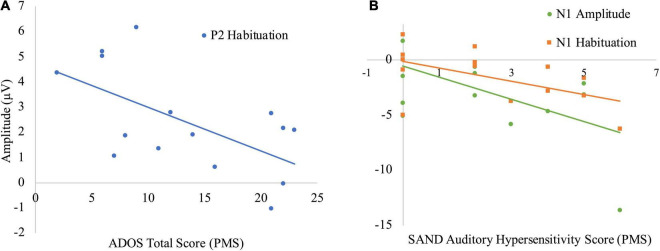
Correlations between electrophysiologic and behavioral measures in PMS. **(A)** Negative correlation between P2_1–2_ habituation and ADOS total score. **(B)** Negative correlation between N1_1_ amplitude and SAND auditory hypersensitivity, as well as N1_1–2_ habituation and SAND auditory hypersensitivity.

A significant negative Pearson correlation was found in the PMS group between the P2_1_ amplitude and ADOS-2 Restricted and Repetitive Behaviors (RRB) score (*r* = −0.60, *p* = 0.02), wherein lower P2 response was associated with higher levels of repetitive behaviors. There was also a significant negative relationship between the P2_1–2_ habituation and the ADOS-2 RRB (*r* = −0.67, *p* = 0.01), Social Affect (SA) (*r* = −0.55, *p* = 0.03) and Total scores (*r* = −0.60, *p* = 0.02), with more ASD symptoms associated with reduced P2 habituation. No other electrophysiological measures were correlated with ADOS-2 scores. See Figures 3, 4A.

#### Sensory Assessment for Neurodevelopmental Disorders

The total SAND scores as well as the three subdomains of the auditory domain (hypersensitivity, hyposensitivity, and seeking) were selected for analysis given our auditory task. The total, auditory hyposensitivity, and auditory seeking scores did not correlate with any of the ERP response or habituation metrics. However, the auditory hypersensitivity domain correlated with both the N1_1_ amplitude (*r* = −0.54, *p* = 0.04) and N1_1–2_ habituation (*r* = −0.54, *p* < 0.05); greater auditory hypersensitivity was associated with both larger initial N1 response and greater N1 habituation. See Figures 3, 4B.

## Discussion

In this study, we investigated electrophysiological markers of early auditory processing in groups of individuals with PMS, iASD, and NT controls. Our findings demonstrate preliminary evidence of intact response and habituation to simple auditory stimuli in PMS. Indeed, across both iASD and PMS, we found robust responses to initial tones, followed by a decay of response to the second tone and relatively asymptotic responses to the third and fourth tones. This response pattern corresponds with what has been shown in canonical habituation tasks in healthy controls (Budd et al., 1998; Ozesmi et al., 2000). These results were surprising, given the ample evidence for heightened sensory sensitivity in ASD (Karhson and Golob, 2016; Hudac et al., 2018), clinical reports of auditory hyporesponsiveness in PMS (Phelan and McDermid, 2012; Mieses et al., 2016), as well as findings in *Shank3* animal models showing reduced auditory startle (Drapeau et al., 2018). Latency of neural response to tones also did not differ among groups, despite prior studies in iASD showing slowed response to auditory stimuli (Roberts et al., 2010) and others in animal models of PMS showing delayed response to auditory stimulation (Engineer et al., 2018). This divergence of the electrophysiological phenotypes of humans with PMS and equivalent animal models should be explored further to identify whether there are physiological differences driving the discrepancy.

With respect to PMS, our findings, though exploratory given the modest sample size, suggest that despite evidence for diminished behavioral response to auditory stimuli (Phelan and McDermid, 2012; Mieses et al., 2016), a key neural aspect of early auditory processing may be intact. A study that investigated the neurocognitive perception of communicative sounds using functional magnetic resonance imaging (fMRI) found similarly intact neural responses in PMS (Wang et al., 2016). Together, these results point toward unaffected early cortical processing of sounds in this population. If this is the case, alterations in *later* higher order stages of processing and the subsequent interpretation of auditory information that are beyond our measurement may contribute to the sensory phenotype of PMS. Such higher order cognitive processes could include extracting the relevance and meaning of stimuli, directing or sustaining auditory attention, and utilizing auditory input to direct behavior.

Our findings in PMS are distinct from those observed in FXS, where reduced habituation of the N1 was detected in a comparable sample size (Ethridge et al., 2016). Interestingly, the sensory phenotype of PMS and FXS differ; in PMS there is general sensory *hypo*sensitivity (Mieses et al., 2016), but FXS is characterized by sensory *hyper*sensitivity (Ethridge et al., 2019). Dissociable neural response patterns to auditory stimulation may indicate that the neuropathology of PMS and FXS diverge on a fundamental level. Whereas FXS demonstrates alterations in early cortical processing deficits, it may be that the sensory symptoms characteristic of PMS are perhaps driven instead by issues with interpreting and attending to sensory information or initiating an appropriate behavioral response. This observed preservation of early auditory habituation parallels pre-clinical findings in at least one *Shank3* mouse model that found no disruption of sensory gating (Drapeau et al., 2018). These results serve as a contrast to findings of reduced electrophysiological amplitude in response to visual stimuli in individuals with PMS (Siper et al., 2021), suggesting dissociation of cortical processing across at least these two sensory domains. Future research should also assess whether cortical responses to somatosensory stimulation resemble vision and parallel the behavioral tactile hyposensitivity found in PMS or if they are incongruent as with audition. This work on additional sensory modalities will help clarify the extent to which the neural bases of sensory processing abnormalities overlap or diverge across modalities in PMS.

Though we did not see overall group differences between PMS and NT controls, patterns of auditory response and habituation did nominally correlate with both genotype and phenotype. Class II deletions were associated with reduced initial P2 response and poorer habituation of the N1 response to repeated tones, indicating potential dissociation based on extent of genetic deletion. Likewise, associations between the ADOS-2 and electrophysiological measures indicate a possible interaction between deletion size and ASD traits that could be explored further in larger samples. Finally, higher levels of clinically-observed auditory hypersensitivity were nominally associated with both larger N1 amplitudes to the initial tone and greater subsequent N1 habituation to the second tone. This pattern of greater habituation to sounds that initially evoke a particularly large response may result in behavioral hyposensitivity over time to sounds such as tones, or manifest as aversion responses. If it also extends to complex auditory input like speech or to socially-relevant sounds (e.g., having one’s name called), it could be a contributor to reduced social engagement reported in PMS (Burdeus-Olavarrieta et al., 2021). With regard to the correlation with genotype, as the larger deletion sizes entail additional missing genetic material, it may be that other genes beyond *SHANK3* contribute to auditory habituation differences. These results must be interpreted cautiously and should be investigated in larger cohorts.

Finally, our findings of similar early processing of auditory habituation in groups with iASD as compared to both PMS and NT controls despite notably different sensory phenotypes imply that the level at which auditory processing diverges in these group is likely not in the most basic response and habituation to repeated simple tones. Recent studies of auditory habituation in ASD continue to yield inconsistent results, with some finding reduced habituation (Gandhi et al., 2020; Jamal et al., 2020) while others, like us, finding no differences between ASD and control groups (Top et al., 2018; Kuiper et al., 2019). Collectively, these studies exemplify the heterogeneous nature of ASD and how differences in samples and experimental parameters can influence results. The genetics-first approach used in this study offers a promising way to parse this heterogeneity and pave the way for greater understanding of sensory differences along the autism spectrum.

## Limitations

Several limitations of this study stem from inherent issues with studying a rare genetic disorder, which makes recruitment, matching subjects, as well as obtaining large sample size and/or developmentally narrow age cohorts and high power results difficult. Although this research utilized a respectable sample size for an experimental study involving a rare disorder, a broader age range than is typical of most EEG studies also was needed to adequately sample the PMS population. The number of participants also remains small relative to typical EEG study samples. This small sample size also meant that we could not correct for multiple comparisons in correlations analyses, as overly stringent significance criteria in a sample of our size would be at high risk for obscuring small but meaningful results. As such, we report and discuss nominally significant findings to inform avenues for future work; replication in larger, independent samples is certainly needed.

Additional limitations stem from the complexity of our cross-group comparisons. First, groups varied considerably in terms of sex distribution; given limited research on the effect of sex on sensory processing (Osório et al., 2021), sex differences could be confounding. However, by and large, we saw few group differences for sex to have inadvertently driven. Second, we do not have IQ information for our NT group and our PMS sample had a significantly lower cognitive level than the iASD sample. However, at least within our PMS group, IQ did not correlate with any of the electrophysiological measures, and this observation is consistent with past research on IQ and sensory processing in ASD, which suggests a negligible – or at best inconsistent - relationship between the two domains (Rogers et al., 2003; Crane et al., 2009; Kargas et al., 2015; Sanz-Cervera et al., 2015). Thus, though intellectual level was not matched across groups, both our findings and past research supports that low-level detection of auditory information is decoupled from measured intelligence. Third, though our iASD sample is modest, particularly in light of the heterogeneity across ASD, our electrophysiological findings in iASD vs NT replicates earlier work (Kohl et al., 2014; Takahashi et al., 2016).

Finally, our PMS group was somewhat unusual in that only thirty three percent of our PMS sample also had an ASD diagnosis. This percentage is low given the higher prevalence typically reported in the literature (Kolevzon et al., 2014), though we note that ASD diagnosis did not interact with electrophysiological results in our PMS sample. Nonetheless, our results may not be generalizable to the PMS population as a whole, albeit given the ascertainment bias associated with genetic testing, it is possible that true ASD rates are lower than estimated among known cases. As genetic testing becomes less expensive and more widespread, additional diagnoses of PMS are expected to be made, enabling studies with larger sample sizes and smaller age ranges, as well as better matching based on functioning level, ASD diagnosis, comorbidities, and sensory phenotype. At that time, studies may be able to more clearly dissociate the neurophysiology of ASD, PMS with ASD, and PMS without ASD to better understand the interaction. At present, however, this study is worthwhile in offering the first-ever electrophysiological experimental look into brain processes subserving auditory function in the PMS population. Our findings of intact function are intriguing, particularly given the severity of deficits in this population.

## Conclusion

This study is among the first to explore the neurocognitive basis of the PMS sensory phenotype and serves as the basis for future auditory neurophysiology work in this population. The preserved initial electrophysiological functioning shown here suggests that alterations in downstream processing of sounds, which deals more with extracting the relevance and meaning of stimuli and guiding behavioral response, may drive the hyporesponsive phenotype in PMS. Relations between the electrophysiological measures and ASD diagnosis, symptoms, and 22q13 deletion size in PMS point toward individual variability and genotype-phenotype relationships. Future studies that probe both early and late auditory processing in larger samples of people with PMS would help elucidate the source of these individual differences and identify where in the auditory perceptual pathway the breakdown in processing occurs. This knowledge may aide in the development of targeted therapeutics that reduce the negative consequences of auditory hyporesponsiveness in this group.

## Data Availability Statement

The raw data supporting the conclusions of this article will be made available by the authors, without undue reservation.

## Ethics Statement

The studies involving human participants were reviewed and approved by the Icahn School of Medicine Program for the Protection of Human Subjects. Written informed consent to participate in this study was provided by the participant or the participant’s legal guardian/next of kin, as appropriate. Verbal assent was also provided by participants with capacity.

## Author Contributions

EI and JF-F designed the study and wrote the manuscript. EI, HG, SG, YZ, SB, and CM participated in data collection and processing. EI, SG, TL, and JF-F contributed to data analysis. DH, PS, AK, and JF-F characterized the participants and provided clinical supervision. SG, TL, PS, JB, and AK provided substantial revisions. All authors read and approved the manuscript.

## Conflict of Interest

The Sensory Assessment for Neurodevelopmental Disorders (SAND) was developed by PS and was licensed by Mount Sinai to Stoelting Co. AK received research support from AMO Pharma and consults to Ovid Therapeutics, GW Pharmaceuticals, Acadia, Alkermes, Ritrova Therapeutics, Jaguar Therapeutics, Neuren Pharmaceuticals, Clinilabs Drug Development Corporation, and Scioto Biosciences. JB consults to BridgeBio, holds a patent for IGF-1 for the treatment of Phelan-McDermid syndrome, holds an honorary professorship from Aarhus University Denmark, receives research support from Takeda and Oryzon, and is a journal editor for Springer Nature. The remaining authors declare that the research was conducted in the absence of any commercial or financial relationships that could be construed as a potential conflict of interest.

## Publisher’s Note

All claims expressed in this article are solely those of the authors and do not necessarily represent those of their affiliated organizations, or those of the publisher, the editors and the reviewers. Any product that may be evaluated in this article, or claim that may be made by its manufacturer, is not guaranteed or endorsed by the publisher.
